# Sad, blue, or depressed days, health behaviors and health-related quality of life, Behavioral Risk Factor Surveillance System, 1995–2000

**DOI:** 10.1186/1477-7525-2-40

**Published:** 2004-07-30

**Authors:** Rosemarie Kobau, Marc A Safran, Matthew M Zack, David G Moriarty, Daniel Chapman

**Affiliations:** 1 Health Care and Aging Studies Branch, Division of Adult and Community Health, National Center for Chronic Disease Prevention and Health Promotion, Centers for Disease Control and Prevention, 4770 Buford Highway NE, Mailstop MS K-51, Atlanta, GA 30341, USA; 2 Office of the Director, National Center for HIV, STD, and TB Prevention, Centers for Disease Control and Prevention, Atlanta, GA, USA; 3 Emerging Investigations and Analytical Methods Branch, Division of Adult and Community Health, National Center for Chronic Disease Prevention and Health Promotion, Centers for Disease Control and Prevention, Atlanta, GA, USA

## Abstract

**Background:**

Mood disorders are a major public health problem in the United States as well as globally. Less information exists however, about the health burden resulting from subsyndromal levels of depressive symptomatology, such as feeling sad, blue or depressed, among the general U.S. population.

**Methods:**

As part of an optional Quality of Life survey module added to the U.S. Behavioral Risk Factor Surveillance System, between 1995–2000 a total of 166,564 BRFSS respondents answered the question, "During the past 30 days, for about how many days have you felt sad, blue, or depressed?" Means and 95% confidence intervals for sad, blue, depressed days (SBDD) and other health-related quality of life (HRQOL) measures were calculated using SUDAAN to account for the BRFSS's complex sample survey design.

**Results:**

Respondents reported a mean of 3.0 (95% CI = 2.9–3.1) SBDD in the previous 30 days. Women (M = 3.5, 95% CI = 3.4–3.6) reported a higher number of SBDD than did men (M = 2.4, 95% CI = 2.2–2.5). Young adults aged 18–24 years reported the highest number of SBDD, whereas older adults aged 60–84 reported the fewest number. The gap in mean SBDD between men and women decreased with increasing age. SBDD was associated with an increased prevalence of behaviors risky to health, extremes of body mass index, less access to health care, and worse self-rated health status. Mean SBDD increased with progressively higher levels of physically unhealthy days, mentally unhealthy days, unhealthy days, activity limitation days, anxiety days, pain days, and sleepless days.

**Conclusion:**

Use of this measure of sad, blue or depressed days along with other valid mental health measures and community indicators can help to assess the burden of mental distress among the U.S. population, identify subgroups with unmet mental health needs, inform the development of targeted interventions, and monitor changes in population levels of mental distress over time.

## Background

Mood disorders are a major public health problem in the United States as well as globally, imposing a substantial burden of disability, impaired quality of life, and death if they remain untreated [1-3]. National estimates for 12-month prevalence of depressive disorders for adults aged 18 and over range between 6.3%-11.3% depending on the assessment tools, criteria used, and populations studied [1,4-6]. The lifetime prevalence of six selected mood disorders, including major depressive episode, dysthymia, and bipolar disorder as assessed by the Diagnostic Interview Schedule [7] among 7,667 respondents aged 17–39 years to the third National Health and Nutrition Examination Survey, was 8.6% for major depressive episode; 7.7% for severe major depressive episode; 6.2% for dysthymia; 3.4% for combined major depressive episode and dysthymia, 1.6% for any bipolar disorder, and 11.5% for any mood disorder [2]. In the Alameda County Study, 6.6% of men and 10.1% of women aged 50 years or older met DSM-III-R and DSM IV [8,9] symptom criteria for major depression within the past two weeks [10]. By 2020, depression will become the second leading cause for disease burden [11]

Mental health disorders due to depression, anxiety and substance use are not only burdensome by themselves, but they can complicate existing physical disorders and also increase risk for other physical comorbidity [3,12,13]. For example, psychological distress might interfere with medication adherence for an existing disorder such as hypertension, but also increase the likelihood of adopting unhealthy behaviors such as smoking, excessive alcohol use, or overeating that can further impair physical health. Both major depressive disorder and subsyndromal levels of depression are associated with similar demographic, social, psychiatric and physical health predictors [14,15]. Results from the 1980–1985 Epidemiologic Catchment Area Study indicated that almost 30% of the population reported having experienced a period lasting at least two weeks in their lifetime when they felt sad, blue or depressed or lost interest in previously pleasurable things or activities [16]. The inclusion of lesser levels of depressive symptomatology when calculating estimates of the prevalence of diagnosable depression could inflate such estimates [17]. However, an examination of subsyndromal levels of depression to determine what proportion of the population is at risk for major depressive disorder can be useful for communities interested in preventing depression [12]. Examining subsyndromal depression and its associated risk with unhealthful behaviors, furthermore, can highlight associations between feeling sad, blue or depressed and behaviors risky to health [3], and is of public health interest [12,18-20].

For at least one year since 1995, more than one-third of state health departments have assessed the number of recent days that adults "felt sad, blue or depressed" using the Behavioral Risk Factor Surveillance System (BRFSS). This measure has good construct validity when compared with other BRFSS health-related quality-of-life (HRQOL) domains related to mental health [21], and has acceptable reliability and criterion validity when compared with the mental health scales of the Medical Outcomes Study Short-Form 36 (SF-36) [22] and with the Center for Epidemiologic Studies Depression Scale (CES-D) [23] among older, low-income African-American men [24].

Using a large multi-state sample, this study is the first to focus on the prevalence of self-reported "sad, blue or depressed" days (SBDD) overall and in sociodemographic subgroups in the United States. It also examines the construct validity of the measure.

## Methods

The BRFSS, which is designed to monitor behavioral health risks in the United States, is an annual random-digit-dialed telephone survey of the non-institutionalized civilian population aged 18 years or older conducted in all states and the District of Columbia [25]. As part of an optional Quality of Life survey module that was added to the BRFSS and used in 38 states and the District of Columbia in one or more years from 1995 through 2000, a total of 166,564 BRFSS respondents answered the question, "During the past 30 days, for about how many days have you felt sad, blue, or depressed?" The module also contains questions on the number of recent days of pain, anxiety, sleeplessness and on other HRQOL domains.

Respondents answered standard BRFSS questions about age, race/ethnicity, education, employment, income, marital status, health status, physical health, mental health, activity limitation, access to care, and the presence of certain health conditions such as hypertension and diabetes. Respondents also answered questions about how often they engaged in behaviors risky to health, such as smoking and binge drinking, and in health promoting behaviors, such as using a seat belt and exercising. Each respondent's body mass index (BMI), (weight in kilograms divided by the square of height in meters), was classified according to the National Institutes of Health criteria as either underweight (<18.5 kg/m^2^), normal weight (18.5< 25.0 kg/m^2^), overweight (25.0 to < 30.0 kg/m^2^), or obese (≥ 30.0 kg/m^2^) [26].

Individual responses were weighted to reflect the age and sex distribution of each state's population during each survey year. To account for the BRFSS's complex sample survey design, means (M) and 95% confidence intervals (CI) for SBDD and other HRQOL measures were calculated using SUDAAN (Research Triangle, release 8.0.0, Research Triangle Park, NC: 2001). Because mean SBDD varied by five-year age groups, the analyses were directly standardized to the age distribution of adults aged 18 years or older from the 2000 U.S. Census to control for confounding by age.

## Results

Respondents reported a mean of 3.0 (95% CI = 2.9–3.1) SBDD in the previous 30 days. About 43.4% of respondents reported one or more SBDD including 7.9% who reported 14 or more SBDD. Women (M = 3.5, 95% CI = 3.4–3.6) reported a higher number of SBDD than did men (M = 2.4, 95% CI = 2.2–2.5) (Table 1). Young adults aged 18–24 years reported the highest number of SBDD, whereas older adults aged 60–84 reported the fewest, with the gap in mean SBDD between men and women decreasing with increasing age (Table 1).

**Table 1:** Mean number of days U.S. adults felt sad, blue or depressed (Behavioral Risk Factor Surveillance System)*

	**Males**			**Females**			**Males & Females**		
	N	Mean	95% CI	N	Mean	95% CI	N	Mean	95% CI

Demographic, behavioral risk group variable									

**5 year age group**									
18–19 yrs.	1,704	2.8	2.4–3.2	1,914	4.5	4.0–5.0	3,618	3.6	3.2–3.9
20–24 yrs.	4,899	2.8	2.5–3.0	6,697	4.0	3.7–4.2	11,596	3.4	3.2–3.6
25–29 yrs	6,439	2.4	2.2–2.6	8,881	3.4	3.2–3.6	15,320	2.9	2.7–3.1
30–34 yrs.	7,163	2.2	2.0–2.4	10,016	3.6	3.4–3.8	17,179	2.9	2.7–3.0
35–39 yrs.	7,763	2.5	2.3–2.6	11,061	3.9	3.7–4.1	18,824	3.2	3.0–3.3
40–44 yrs.	7,589	2.6	2.4–2.8	10,430	3.7	3.5–3.9	18,019	3.2	3.0–3.3
45–49 yrs.	6,729	2.7	2.5–2.9	9,297	3.8	3.5–4.0	16,026	3.2	3.0–3.4
50–54 yrs.	5,722	2.6	2.4–2.9	7,986	3.8	3.5–4.1	13,708	3.2	3.0–3.4
55–59 yrs.	4,525	2.2	1.9–2.4	6,424	3.6	3.3–3.9	10,949	3.0	2.7–3.2
60–64 yrs.	3,806	2.2	1.9–2.6	5,489	3.2	2.9–3.4	9,295	2.7	2.5–2.9
65–69 yrs.	3,733	1.8	1.6–2.1	5,905	2.6	2.4–2.9	9,638	2.3	2.1–2.5
70–74 yrs.	3,232	1.9	1.6–2.2	5,629	2.8	2.5–3.1	8,861	2.4	2.2–2.6
75–79 yrs.	2,287	2.2	1.8–2.5	4,627	2.8	2.5–3.1	6,914	2.5	2.3–2.8
80–84 yrs.	1,270	2.3	1.8–2.8	2,949	2.6	2.3–3.0	4,219	2.5	2.2–2.8
85+ yrs.	616	2.4	1.7–3.1	1,782	3.1	2.6–3.7	2,398	2.9	2.4–3.4
All categories	67,477	2.4	2.2–2.5	99,087	3.5	3.4–3.6	166,564	3.0	2.9–3.1
**Race/ethnicity**									
Asian/Pacific									
Islander	1,098	1.6	1.2–2.0	1,216	2.5	2.0–2.9	2,314	2.0	1.7–2.3
White	54,502	2.3	2.2–2.4	77,761	3.3	3.3–3.4	132,263	2.8	2.7–2.9
Hispanic	4,395	2.9	2.5–3.2	6,455	4.3	3.9–4.6	10,850	3.6	3.3–3.8
Black	5,852	2.9	2.7–3.2	11,463	4.5	4.2–4.7	17,315	3.8	3.6–4.0
Native American									
Indian/Alaska									
Native	679	3.0	2.3–3.7	956	5.0	4.1–5.9	1,635	4.0	3.4–4.6
Other	431	3.0	2.0–4.0	538	5.2	4.0–6.4	969	4.2	3.2–5.1
All categories	66,526	2.4	2.3–2.5	97,851	3.6	3.4–3.7	165,346	3.0	2.9–3.1
**Education**									
< High school	7,772	3.7	3.4–4.0	12,468	5.6	5.3–5.9	20,240	4.7	4.5–4.9
HS graduate	20,727	2.6	2.4–2.7	32,902	3.8	3.7–3.9	53,629	3.2	3.1–3.3
Some college	17,770	2.4	2.2–2.5	27,700	3.4	3.3–3.5	45,470	2.9	2.8–3.0
College graduate	21,068	1.7	1.6–1.8	25,826	2.4	2.3–2.5	46,894	2.0	1.9–2.1
All categories	67,337	2.4	2.3–2.5	98,896	3.6	3.5–3.7	166,233	3.0	2.9–3.1
**Employment**									
Employed (wages)	41,465	1.9	1.8–2.0	51,567	2.9	2.8–3.1	93,032	2.4	2.3–2.5
Self-employed	7,497	2.3	2.0–2.5	5,354	3.0	2.7–3.3	12,851	2.5	2.3–2.7
Retired	12,054	3.6	2.4–4.9	19,880	2.4	1.5–3.2	31,934	3.0	2.0–4.0
Student	1,997	2.0	1.6–2.4	3,019	3.8	3.1–4.5	5,016	3.3	2.7–3.9
Homemaker	168	2.7	1.6–3.9	11,549	3.5	3.3–3.8	11,717	3.5	3.3–3.7
Unemp. < 1 yr.	1,238	4.5	3.9–5.1	2,094	6.4	5.5–7.3	3,332	5.7	4.9–6.5
Unemp. ≥1 yr.	766	5.3	4.4–6.2	1,657	6.3	5.6–7.0	2,423	6.1	5.5–6.7
Unable to work	2,225	9.6	8.5–10.6	3,878	10.7	10.0–11.5	6,103	10.2	9.5–10.8
All categories	67,410	2.4	2.3–2.5	98,998	3.6	3.5–3.7	166,408	3.0	2.9–3.1
**Income**									
<$15,000	5,194	5.4	5.0–5.8	13,012	6.5	6.2–6.8	18,206	6.1	5.8–6.3
$15,000–$24,999	10,765	3.3	3.1–3.4	18,662	4.5	4.3–4.7	29,427	3.9	3.8–4.0
$25,000–$49,999	23,046	2.2	2.1–2.4	29,419	3.2	3.0–3.3	52,465	2.7	2.6–2.8
≥$50,000	20,046	1.7	1.6–1.8	21,382	2.4	2.3–2.6	41,428	2.0	1.9–2.1
All categories	59,051	2.5	2.4–2.6	82,475	3.6	3.5–3.7	141,526	3.0	2.9–3.1
**Marital status**									
Currently married	38,756	2.1	1.8–2.3	49,671	3.0	2.8–3.1	88,427	2.5	2.4–2.6
Never married	14,429	3.0	2.8–3.2	15,847	3.6	3.3–3.8	30,276	3.3	3.1–3.4
Divorced	8,060	3.6	3.1–4.2	13,600	5.0	4.7–5.2	21,660	4.4	4.1–4.8
Unmarried couple	1,536	3.8	2.6–5.0	2,008	4.7	3.7–5.6	3,544	4.5	3.6–5.5
Widowed	2,980	4.2	3.4–5.0	14,562	6.6	5.5–7.7	17,542	6.0	5.1–6.9
Separated	1,609	5.4	4.6–6.1	3,164	6.3	5.7–6.9	4,773	6.0	5.5–6.5
All categories	67,370	2.4	2.3–2.5	98,852	3.6	3.5–3.6	166,222	3.0	2.9–3.1
**Participate in any physical activity**									
Yes	36,039	2.1	2.0–2.2	49,472	3.1	3.0–3.2	85,511	2.6	2.5–2.7
No	13,388	3.1	3.0–3.3	22,607	4.6	4.4–4.8	35,995	3.9	3.8–4.1
All categories	49,427	2.4	2.3–2.5	72,079	3.6	3.5–3.7	121,506	3.0	2.9–3.1
**Body mass index**									
Underweight	608	4.6	3.6–5.6	3,192	4.5	4.0–5.0	3,800	4.5	4.1–5.0
Normal	23,561	2.5	2.4–2.6	47,187	3.0	2.9–3.1	70,748	2.8	2.7–2.9
Overweight	30,305	2.1	2.0–2.2	25,987	3.8	3.6–3.9	56,292	2.7	2.6–2.8
Obese	12,295	2.8	2.6–2.9	17,031	5.0	4.7–5.2	29,326	3.8	3.7–4.0
All categories	66,769	2.4	2.3–2.5	93,397	3.6	3.5–3.7	160,166	3.0	3.0–3.1
**Ever drank ≥ 5 drinks in past 30 days at once**									
Yes	6,403	2.6	2.4–2.9	2,968	5.0	4.4–5.5	9,371	3.3	2.9–3.6
No	18,206	2.2	2.0–2.3	29,554	3.3	3.2–3.4	47,760	2.8	2.7–2.9
All categories	24,609	2.3	2.2–2.4	32,522	3.4	3.3–3.5	57,131	2.8	2.7.2.9
**Smoking status**									
Never smoked	31,088	1.9	1.8–2.0	57,611	3.0	2.9–3.1	88,699	2.6	2.4–2.7
Former smoker	18,973	2.2	2.1–2.4	19,639	3.5	3.4–3.7	38,612	2.9	2.8–3.0
Smokes <1 pack/day	7,112	3.1	2.8–3.3	11,357	5.0	4.7–5.2	18,469	4.1	3.9–4.3
Smokes ≥1 pack/day	8,751	4.2	3.8–4.6	8,688	6.1	5.7–6.4	17,439	5.0	4.7–5.2
All categories	65,924	2.4	2.3–2.5	97,295	3.6	3.4–3.7	163,219	3.0	2.9–3.1
**Seatbelt use**									
Always	7,960	2.2	2.0–2.4	13,769	3.1	2.9–3.2	21,729	2.7	2.6–2.8
Nearly always	2,142	2.0	1.7–2.3	2,469	3.5	3.1–3.9	4,611	2.7	2.4–2.9
Sometimes	1,287	2.8	2.4–3.3	1,291	4.4	3.8–4.9	2,578	3.5	3.1–3.8
Seldom	698	3.1	2.4–3.8	578	5.5	4.5–6.4	1,276	3.9	3.4–4.5
Never	901	4.0	3.3–4.7	639	6.1	5.0–7.1	1,540	4.7	4.1–5.3
All categories	12,988	2.4	2.3–2.5	18,746	3.4	3.2–3.5	31,734	2.9	2.8–3.0
**Time when could not afford to get medical care in past year**									
Yes	5,629	5.4	5.0–5.7	11,867	6.8	6.5–7.1	17,496	6.2	6.0–6.5
No	61,740	2.1	2.0–2.2	87,069	3.1	3.0–3.2	148,809	2.6	2.5–2.7
All categories	67,369	2.4	2.3–2.5	98,936	3.6	3.4–3.7	166,305	3.0	2.9–3.1
**Have any kind of health plan?**									
Yes	58,645	2.2	2.1–2.3	87,693	3.3	3.2–3.4	146,338	2.8	2.7–2.9
No	8,657	3.7	3.4–4.1	11,236	5.1	4.7–5.4	19,893	4.4	4.1–4.6
All categories	67,302	2.4	2.3–2.6	98,929	3.6	3.4–3.7	166,231	3.0	2.9–3.1
**Self-rated health**									
Excellent	16,485	1.4	1.3–1.5	22,518	1.9	1.8–2.0	39,003	1.6	1.5–1.7
Very good	22,980	1.7	1.6–1.8	33,099	2.7	2.6–2.8	56,079	2.2	2.1–2.3
Good	18,980	2.5	2.3–2.6	28,081	3.9	3.7–4.0	47,061	3.2	3.1–3.3
Fair	6,441	4.8	4.5–5.1	11,041	7.2	6.8–7.5	17,482	6.0	5.8–6.3
Poor	2,464	11.3	10.2–12.4	4,154	11.2	10.3–12.1	6,618	11.2	10.5–12.0
All categories	67,350	2.4	2.3–2.5	98,893	3.6	3.4–3.7	166,243	3.0	2.9–3.1

Note. *Selected U.S. states, 1995–2000; all variables except age-groups are age-adjusted.

Asians/Pacific Islanders reported the fewest SBDD (M = 2.0, 95% CI = 1.7–2.3), whereas Hispanics, Blacks, American Indians and Alaska Natives, and non-whites of another race/ethnicity reported about 4–5 SBDD; in the last three of these groups, the gaps between men and women were larger. Adults with more education reported fewer SBDD, with the gap between men and women diminishing with more education. Respondents who were unemployed or unable to work reported more SBDD than the employed. The number of SBDD decreased with increasing levels of annual household income. Widowed or separated adults reported about 6 SBDD, whereas respondents who were currently married reported the fewest number of SBDD (M = 2.5); the gap between men and women was least among those who had never married.

SBDD was associated with an increased prevalence of behaviors risky to health, extremes of BMI, less access to health care, and worse self-rated health status. Respondents who reported physical inactivity, binge drinking, seldom-use or nonuse of seatbelt, or any or more cigarette smoking reported substantially higher numbers of SBDD than those who did not report engaging in these risky behaviors (Table 1). Subjects who were either underweight or obese reported a higher number of SBDD than those of normal weight or overweight. Obese women reported more SBDD (M = 5.0, 95% CI = 4.7–5.2) than obese men (M = 2.8, 95% CI = 2.6–2.9). However, underweight men and women reported the same number (about 4.5) of SBDD. Respondents who could not afford to see a physician at least once during the past year or who had no health care insurance coverage reported more SBDD than those who could afford to see a physician or had such coverage. Although subjects with excellent self-rated health status reported 1.6 SBDD, those with poor health reported 11.2 SBDD, with the largest gap (2.4 days) occurring between men and women with fair health status.

### Additional Construct Validity

SBDD were associated with other physical and mental HRQOL domains in expected ways. Mean SBDD increased with progressively higher levels (i.e., 0, 1–2, 3–13, 14–29, and 30 days) of physically unhealthy days, mentally unhealthy days, unhealthy days, activity limitation days, anxiety days, pain days, and sleepless days (Table 2). Similarly, subjects who reported more days when they felt "very healthy and full of energy" reported fewer SBDD. The gap between men and women was smaller at lower levels of these other HRQOL domains.

**Table 2:** Mean number of days adults felt sad, blue or depressed by HRQOL* and sex**

**HRQOL variable**	**Males**			**Females**			**Male & Females**		
	N	Mean	95% CI	N	Mean	95% CI	N	Mean	95% CI

**Physically unhealthy days**									
0 days	48,188	1.7	1.6–1.8	63,192	2.6	2.5–2.7	111,380	2.2	2.0–2.3
1–2 days	6,792	2.3	2.1–2.5	10,855	3.0	2.8–3.2	17,647	2.6	2.5–2.8
3–13 days	6,371	3.6	3.4–3.9	12,631	4.6	4.4–4.8	19,002	4.2	4.1–4.4
14–29 days	1,959	5.9	5.3–6.5	4,614	7.5	7.0–8.0	6,573	6.9	6.5–7.3
30 days	3,539	8.1	7.3–8.8	6,207	9.3	8.6–9.9	9,746	8.7	8.3–9.2
all categories	66,849	2.4	2.3–2.5	97,499	3.5	3.4–3.6	164,348	3.0	2.9–3.1
**Mentally unhealthy days**									
0 days	50,042	1.1	1.0–1.2	63,446	1.5	1.4–1.6	113,488	1.3	1.2–1.4
1–2 days	5,446	2.1	1.9–2.2	9,644	2.2	2.0–2.3	15,090	2.1	2.0–2.2
3–13 days	6,717	4.9	4.6–5.2	14,219	5.2	5.0–5.3	20,936	5.0	4.8–5.2
14–29 days	2,194	11.1	10.5–11.8	5,318	12.6	12.2–13.1	7,512	12.1	11.7–12.5
30 days	2,366	16.1	15.3–16.9	5,173	17.2	16.6–17.7	7,539	16.8	16.3–17.2
all categories	66,765	2.4	2.3–2.5	97,800	3.5	3.4–3.6	164,565	3.0	2.9–3.1
**Unhealthy days^+^**									
0 days	38,831	0.9	0.8–1.0	45,983	1.3	1.2–1.4	84,814	1.1	1.0–1.2
1–2 days	7,675	1.4	1.3–1.5	11,093	1.5	1.4–1.6	18,768	1.5	1.4–1.6
3–13 days	10,963	2.9	2.8–3.0	20,785	3.4	3.3–3.5	31,748	3.2	3.1–3.5
14–29 days	3,344	6.7	6.3–7.2	7,935	7.7	7.4–8.0	11,279	7.4	7.1–7.6
30 days	5,446	10.7	10.2–11.3	10,678	12.4	12.1–12.8	16,124	11.7	11.4–12.1
all categories	66,259	2.4	2.3–2.5	96,474	3.5	3.4–3.6	162,733	3.0	2.9–3.1
**Activity limitation days**									
0 days	56,202	1.7	1.6–1.8	77,711	2.6	2.5–2.7	133,913	2.1	2.0–2.2
1–2 days	3,556	2.5	2.2–2.7	6,446	3.3	3.1–3.5	10,002	3.0	2.8–3.1
3–13 days	3,462	5.3	4.9–5.7	7,169	6.6	6.3–6.9	10,631	6.1	5.8–6.4
14–29 days	1,305	10.2	9.4–11.0	2,887	11.8	11.2–12.5	4,192	11.2	10.7–11.7
30 days	1,962	12.3	11.3–13.4	3,204	13.6	12.7–14.5	5,166	13.0	12.2–13.7
all categories	66,487	2.4	2.3–2.5	97,417	3.5	3.4–3.6	163,904	3.0	2.9–3.1
**Anxiety days**									
0 days	32,664	0.6	0.5–0.7	38,600	0.8	0.7–0.9	71,264	0.7	0.6–0.8
1–2 days	11,739	1.1	1.0–1.2	17,592	1.3	1.2–1.4	29,331	1.2	1.1–1.3
3–13 days	14,076	3.1	3.0–3.3	25,130	3.7	3.6–3.8	39,206	3.4	3.3–3.5
14–29 days	3,946	9.4	8.9–9.9	7,834	10.2	9.9–10.6	11,780	9.9	9.6–10.1
30 days	4,018	13.9	13.3–14.5	8,017	15.0	14.6–15.5	12,035	14.5	14.2–14.9
all categories	66,443	2.4	2.3–2.5	97,173	3.6	3.4–3.7	163,616	3.0	2.9–3.1
**Pain days**									
0 days	52,475	1.7	1.6–1.8	72,697	2.6	2.5–2.7	125,172	2.2	2.0–2.2
1–2 days	4,421	2.6	2.3–2.8	7,143	3.5	3.3–3.8	11,564	3.1	2.9–3.3
3–13 days	4,815	3.8	3.6–4.1	8,598	5.5	5.2–5.8	13,413	4.8	4.5–5.0
14–29 days	1,762	6.8	6.1–7.4	3,642	8.6	8.1–9.2	5,405	7.9	7.4–8.3
30 days	3,363	9.1	8.3–9.9	5,612	10.3	9.6–11.0	8,975	9.7	9.2–10.3
all categories	66,836	2.4	2.3–2.5	97,692	3.6	3.4–3.7	164,528	3.0	2.9–3.1
**Sleeplessness days**									
0 days	24,107	1.6	1.5–1.7	31,809	2.2	2.1–2.3	55,916	1.9	1.8–2.0
1–2 days	7,750	1.1	1.0–1.2	10,410	1.6	1.5–1.8	18,160	1.4	1.3–1.5
3–13 days	20,007	2.1	2.0–2.2	29,594	2.8	2.7–2.9	49,601	2.5	2.4–2.6
14–29 days	8,407	5.0	4.6–5.3	13,633	6.0	5.8–6.3	22,040	5.5	5.3–5.7
30 days	6,347	6.4	6.0–6.8	12,087	7.9	7.6–8.3	18,434	7.3	7.0–7.6
all categories	66,618	2.4	2.3–2.5	97,533	3.6	3.4–3.7	164,151	3.0	2.9–3.1
**Vitality days**									
0 days	6,247	7.1	6.6–7.5	11,536	9.0	8.6–9.4	17,783	8.2	7.9–8.5
1–2 days	1,450	5.9	5.2–6.6	3,118	7.4	6.8–8.0	4,568	6.8	6.4–7.3
3–13 days	8,912	4.2	4.0–4.4	14,809	5.4	5.2–5.6	23,721	4.9	4.7–5.0
14–29 days	28,280	1.7	1.6–1.8	41,496	2.5	2.3–2.6	69,776	2.1	2.0–2.2
30 days	20,990	1.1	0.9–1.2	24,687	1.3	1.2–1.4	45,677	1.2	1.1–1.3
all categories	65,879	2.4	2.3–2.5	95,646	3.6	3.4–3.7	161,525	3.0	2.9–3.1

Note. *Health-Related Quality of Life (HRQOL) questions are: Now thinking about your physical health which includes physical illness and injury, for how many days in the past 30 days was your physical health not good?; Now thinking about your mental health which includes stress, depression, and problems with emotions, for how many days in the past 30 days was your mental health not good?; During the past 30 days, for about how many days did poor physical or mental health keep you from doing your usual activities, such as self-care, work, or recreation?; During the past 30 days, for about how many days did pain make it hard for you to do your usual activities, such as self-care, work, or recreation?; During the past 30 days, for about how many days have you felt worried, tense, or anxious?; During the past 30 days, for about how many days have you felt you did not get enough rest or sleep?; During the past 30 days, for about how many days have you felt very healthy and full of energy? **Behavioral Risk Factor Surveillance System; Selected U.S. states 1995–2000; all variables are age-adjusted. ^+^A calculated measure which results from the sum of physically unhealthy days and mentally unhealthy days with a maximum of 30 unhealthy days for an individual.

## Discussion

In our study, U.S. adults reported an average of about 3 days during the past 30 days when they felt "sad, blue or depressed." Our results are consistent with previous studies documenting the increased prevalence of depressive symptoms among the following groups: women [27-31], in certain minority racial and ethnic groups [32], people with lower levels of education and income [32,33], people of lower employment status [27,34,35], people formerly married or living together but not married [27,36], and in those with limited or no access to health care [32,37,38]. The gap in the number of SBDD between men and women was less pronounced as socioeconomic status improved. Respondents who reported a higher number of SBDD also reported engaging in unhealthy behaviors such as cigarette smoking, binge drinking, and physical inactivity. Underweight and obese adults also reported higher numbers of SBDD than did normal or overweight adults. These findings extend previous public health studies that have documented an association between self-reported mental distress and behaviors risky to health [39-42].

Given the cross-sectional design of the BRFSS, we were unable to determine whether risky behaviors preceded or followed SBDD. Nonetheless, our findings provide additional evidence for the association of considerable public health importance between negative mood and unhealthful behaviors [43]. For example, in the prospective Stirling County Study (1952–1992), subjects who became depressed were more likely to initiate smoking, continue smoking, and refrain from quitting smoking than those who had never become depressed [44].

Negative mood adversely influences self-efficacy to adopt and maintain healthful behaviors and may thwart other self-motivating processes (e.g., attitudes, outcome expectations, and goals) associated with engaging in healthful behaviors [45]. Perceived inefficacy can foster additional despondency. This finding has implications for public health interventions. For example, psychosocial interventions that elicit positive emotions, instill confidence in adopting health-promoting behavior, and improve people's coping skills might be more effective for individuals with despondent mood than interventions designed to arouse fear regarding the consequences of engaging in risky behaviors–which can foster inefficacy and increased despondency [45].

Our findings support the construct validity of the SBDD measure in this study because SBDD were associated with other physical and mental HRQOL domains in expected ways. Groups with progressively higher numbers of physically unhealthy days, activity limitation days, and pain days reported a higher number of SBDD. Moreover, these associations were more pronounced with mentally unhealthy days and anxiety days, than with physically unhealthy days, activity limitation days, and pain days. We found an exception to the linear relationship between SBDD and HRQOL measures with our measure for sleeplessness. Adults who reported 1–2 days of sleeplessness reported fewer SBDD than those who reported no days of sleeplessness. Sleep disturbance, both insomnia and hypersomnia are symptoms of depression. Those reporting no days of sleeplessness, but more SBDD, might be those with hypersomnia. Additional studies are warranted to examine this hypothesis.

Besides the cross-sectional design, this study has other limitations. Only 38 states and the District of Columbia included the HRQOL supplemental module that assessed SBDD. All states and the District of Columbia, however examined mentally unhealthy days–the number of days respondents experienced poor mental health due to stress, depression or problems with emotions. Mean mentally unhealthy days in the states that assessed SBDD with the HRQOL supplemental module did not differ significantly from that in states that did not. Given the positive correlation between mentally unhealthy days and SBDD (r = 0.6), states that did not assess SBDD would most likely report similar SBDD as states that did include this measure, suggesting similar study results had all states assessed SBDD. Second, BRFSS excludes people who do not have telephones, live in institutions, and persons younger than 18 years. Third, BRFSS may under represent the severely impaired because functional capacity is required to participate in BRFSS. Including this group however, would probably only strengthen the associations we found because the variability of SBDD would increase because the severely impaired would be more likely to report more SBDD. Finally, because our findings on SBDD are based on respondents' self-reports rather than on professionally administered psychiatric evaluations, people who experience SBDD may differ from people with clinical depression.

## Conclusion

The 1999 Surgeon General's report states that mental health and mental illness "are not polar opposites but may be thought of as points on a continuum" [1]. Although most people who report feeling sad, blue or depressed several days each month probably do not have a diagnosable mental disorder, those above a certain threshold of SBDD might be at increased risk for mental illness and physical illness. Additional studies that examine this hypothesis are warranted. Findings from this study, moreover, highlight the relationship between feeling sad, blue or depressed and engaging in risky behaviors, thereby suggesting the need for appropriately designed interventions specifically targeted to a person's individual and social context [18]. Use of this measure of "sad, blue or depressed days" along with other valid mental health measures can help to assess the burden of population mental distress, identify subgroups with unmet mental health needs, inform the development of targeted interventions, and monitor changes in population levels of mental distress over time [12].

Future research might examine in more detail the associations among SBDD, anxiety, vitality, and sleeplessness and their ability to assess mood, anxiety, and sleep disorders. It would also be useful to examine the prevalence and demographic characteristics of those who report 14 or more SBDD, and the criterion validity of this measure with other screening instruments and clinical assessments. While SBDD does not provide a strict measure of diagnosable depression as would validated screening and diagnostic assessments, SBDD and other measures such as activity limitations, alcohol or substance abuse, physical inactivity, and employment status can be useful community indicators for addressing the prevention and treatment of depressive symptoms and associated comorbidity [12].

## Abbreviations

SBDD sad, blue or depressed days

HRQOL health-related quality of life

BRFSS Behavioral Risk Factor Surveillance System

CDC Centers for Disease Control and Prevention
